# Hierarchical vector auto-regressive models and their applications to multi-subject effective connectivity

**DOI:** 10.3389/fncom.2013.00159

**Published:** 2013-11-12

**Authors:** Cristina Gorrostieta, Mark Fiecas, Hernando Ombao, Erin Burke, Steven Cramer

**Affiliations:** ^1^Department of Statistics, University of California at IrvineIrvine, CA, USA; ^2^Department of Statistics, University of WarwickCoventry, UK; ^3^Department of Anatomy and Neurobiology, University of California at IrvineIrvine, CA, USA; ^4^Department of Neurology, University of California at IrvineIrvine, CA, USA

**Keywords:** brain effective connectivity, elastic net, functional magnetic resonance imaging, hierarchical models, multivariate time series, stroke, vector auto-regressive model

## Abstract

Vector auto-regressive (VAR) models typically form the basis for constructing directed graphical models for investigating connectivity in a brain network with brain regions of interest (ROIs) as nodes. There are limitations in the standard VAR models. The number of parameters in the VAR model increases quadratically with the number of ROIs and linearly with the order of the model and thus due to the large number of parameters, the model could pose serious estimation problems. Moreover, when applied to imaging data, the standard VAR model does not account for variability in the connectivity structure across all subjects. In this paper, we develop a novel generalization of the VAR model that overcomes these limitations. To deal with the high dimensionality of the parameter space, we propose a Bayesian hierarchical framework for the VAR model that will account for both temporal correlation within a subject and between subject variation. Our approach uses prior distributions that give rise to estimates that correspond to penalized least squares criterion with the elastic net penalty. We apply the proposed model to investigate differences in effective connectivity during a hand grasp experiment between healthy controls and patients with residual motor deficit following a stroke.

## 1. Introduction

The analysis of brain networks has played an important role in characterizing and understanding brain processes and diseases (Bassett and Bullmore, 2009). Pollonini et al. (2010) showed that connectivity between different regions of the brain can differentiate between subjects with autism from healthy controls, and they suggested that connectivity patterns may provide an indicator for the early detection of autism. Wu et al. (2010) showed that the effective connectivity between the motor regions changes as movements become more automatic in patients with Parkinson's disease. Wang et al. (2011) showed that HIV infection has an effect on resting-state connectivity. Benetti et al. (2009) and Skudlarski et al. (2010) showed the effects of schizophrenia on brain connectivity. Because of the clinical implications of connectivity studies, it is imperative to advance the statistical methodologies for connectivity analyses. In this paper, we develop the hierarchical vector-autoregressive (VAR) model to study alterations in brain effective connectivity in patients with chronic stroke. We shall demonstrate that the hierarchical VAR has a number of advantages: (i) it offers a flexible statistical framework for comparing connectivity across experimental conditions (e.g., active vs. rest) and subject groups (e.g., healthy vs. disease); (ii) it quantifies the extent to which a covariate (such as age or genotype) can modify or moderate connectivity; and (iii) it correctly accounts for between-subject heterogeneity in the connectivity structure by including subject-specific parameters in the connectivity model.

Brain networks are often characterized by two types of connectivity, namely, functional connectivity and effective connectivity, broadly defined in Friston (2004) as follows: functional connectivity is the temporal correlation between remote neurophysiological events while effective connectivity evaluates the influence that one neural system exerts over another. Functional connectivity is typically quantified by the cross-correlation between the time courses obtained from regions of interest (ROIs), but does not explore any lead-lag relationships between these ROIs. In this paper, we focus on effective connectivity, studying lead-lag relationships where the directionality is determined by the temporal sequence in the model.

The statistical approaches that provide information about the directionality of associations are structural equation models (SEM) (Mclntosh and Gonzalez Lima, 1994), dynamic causal models (DCM) (Friston et al., 1993) and Granger causality models (GC) (Granger, 1969; Goebel et al., 2003; Roebroeck et al., 2005). DCM attempts to infer the temporal sequence of events, and possibly non-linear dependence, at the neuronal level. In DCM, one estimates the dependence and directionality of the neural source at the millisecond level using functional magnetic resonance imaging (fMRI) data which is observed every 1–2 s. To achieve inference on the temporal dynamics of a neural system, DCM must make assumptions that include (a) the specification of a generative model that maps the neuronal level activity to the observed fMRI signal and (b) an *a priori* model network structure describing the anatomical inter-regional structure and the effects of stimulus perturbations over the defined network. There are a number of problems with the DCM approach. It requires heavy computing time and thus the number of candidate models that can be considered for comparison has to be constrained. Moreover, it is difficult to assess the validity of the physiological assumptions and goodness of fit of the estimated dynamics of the neuronal signal because the observed fMRI data is recorded at a time scale (usually 1–2 s) that is coarser than that of the inferred neuronal dynamics. The SEM approach aims to describe the covariance of the observed fMRI data given a predefined structure over a set of selected regions. The model coefficients give information of the expected change in hemodynamic activity of one ROI influenced by a unit change in another ROI. However, with SEM, it is difficult to estimate cyclical connections, i.e., those involving feedback relationships. The main drawback of both SEM and DCM is that they rely on *a priori* specifications of one or several models. Therefore these methods are mainly used to *confirm* a priori hypotheses about a brain network rather than explore or identify network connections. In practice, it is not always possible to have *a priori* information on the structures of the networks. This is especially difficult when the number of nodes (or ROIs) in the network is large.

Under these scenarios, Granger causality via the VAR model is a viable option for exploring condition and covariate-specific effects on effective connectivity from the observable data. We point out, however, that “causality” may not necessarily be physiological. Rather, it is statistical in nature: if the hypothetical model *A*, which uses data in ROI 1 to predict activity at ROI 2, gives a more accurate and precise prediction compared to a hypothetical model *B* which does not include ROI 1, then we say that activity at ROI 1 “Granger-causes” activity at ROI 2 (Granger, 1969). The VAR model is the standard framework for investigating Granger causality. It has been widely applied to different brain signal modalities, including fMRI time courses (e.g., Goebel et al., 2003; Harrison et al., 2003; Roebroeck et al., 2005; Gorrostieta et al., 2011) and EEG time series (e.g., Kaminski and Blinowska, 1991; Prado and Huerta, 2002; Fiecas and Ombao, 2011). However, there are also a number of controversial points related to GC within the VAR framework approach. When it is applied to fMRI signals, VAR models could identify spurious relationships because fMRI signals (observed at a coarse temporal resolution) are convolved and delayed versions of the neuronal signals (unobserved processes that unfold at a millisecond scale). Thus, the VAR cannot identify lags or delays in the neuronal process that are smaller than the temporal resolution of the fMRI data. For example, a true delay of 200 and 500 ms at the neuronal level might not be distinguishable from fMRI data which were sampled every 1 s. In this paper, we emphasize that our proposed hierarchical VAR will be used to assess for Granger causality at the *hemodynamic level* rather than neuronal. Thus, when our analysis suggests that ROI 1 “Granger-causes” ROI 2, we conclude from VAR modeling that the *hemodynamic activity* at ROI 1 “Granger-causes” the hemodynamic activity at ROI 2. We do not make any conclusions about the underlying neuronal dynamics. While brain scientists are often more interested to make inference on neural activity, we point out that connectivity at the hemodynamic level can also yield interesting results. One more restriction of the VAR model is that it can only be applied to time series data with stationary periods, e.g., resting-state fMRI time courses or fMRI experiments with a block design. Thus, in this paper we center our proposed model in the context of two experimental conditions presented during blocks of time. Finally, although the notion of GC is not restricted to VAR models, it is often implemented under that context. Thus, directed links are currently restricted only to *linear* associations. We are now developing extensions of the VAR model to *functional* VAR model which has the potential to capture non-linear types of dependence.

Between-subject variation in brain responses plays an important role in the analysis of brain networks and must be accounted for in the statistical model and inference because it can have a significant impact in the analysis. This, in fact, is one of the challenges in the Human Connectome Project (Van Essen and Ugurbil, 2012). Under DCMs, one can account for this heterogeneity via a random effects analysis. For SEM and VAR models, there is no standard statistical approach for group-level analysis. For the latter, Deshpande et al. (2009) performed group-level inference by combining the *p*-values obtained from individual subjects using Fisher's method (Fisher, 1932) to obtain a single *p*-value. However, this approach does not report the extent of between-subject variability in effective connectivity. A natural approach in multi-subject analysis is to proceed with the estimation of connectivity parameters in two stages: in the first stage subject-specific parameters are estimated and on the second stage between-subject variations in the estimates from the first stage are obtained. The two-stage approach is known in the statistical literature to be sub-optimal because information is lost when summarizing the original vector-valued time series for the each subject with their connectivity parameters. A reduction of information occurs, for example, by the omission of the subject-specific covariance matrix of parameters in the second stage, whose estimate is dependent on the length of the time series. In addition, when we have a large number of potential predictors in the model and the aim is to identify the important predictors, the implementation of the two-stage procedure is not immediate since this procedure does not take into account the large number of parameters. Moreover, the inclusion of a penalization criterion for parameter estimation in the two-stage approach is not direct, since in the first stage each subject would have his own set of selected predictors, potentially different across subjects, and in the second stage special attention should be considered in proposing a summary statistic that takes into account the sparsity in the parameters in the group of subjects. In this paper, we build on the approach in Gorrostieta et al. (2011) where they proposed the mixed-effects VAR model that allowed the effective connectivity structure to vary across subjects. Here, we adopt a Bayesian approach for statistical modeling and inference.

In addition to the group-analysis inference, there are other challenges to modeling effective connectivity using the VAR model. For a brain network with *R* ROIs, one would need *R*^2^ connectivity parameters per time lag in the VAR model. Thus, one important problem with the VAR model is that the number of parameters grows quadratically with the number of ROIs considered in the analysis and linearly with the order of the model. Due to the large number of parameters and the collinearity of the regressors of the VAR model, even in the case where there is pre-defined number of brain regions to be included in the model estimation procedures via ordinary least squares could lead to numerical problems, unstable results, and lack of interpretability. Moreover, fMRI time courses for each subject are recorded for small periods of time for each stimulus in the experiment, leading to relatively short time courses to be used for estimating an effectivity connectivity network per experimental condition. One of the most common approaches to manage the large number of parameters in the VAR model is penalized regression (Valdes-Sosa et al., 2005; Andrew Arnold, 2007; Martínez-Montes et al., 2008; Davis et al., 2012). In the present work, we present a model with an estimation procedure that follows the framework of penalized regression while accounting for between subject variability. Even for the classical linear model only a few methods exist that address both the high-dimensionality of the parameter space and the modeling of subject-variation (Bondell et al., 2010; van de Geer, 2010; Fan and Li, 2012). However, these problems have not been addressed and explored in a VAR model context.

Despite the ability of the regularization methods to handle a relatively large number of parameters, we caution that it should not give the false sense of security to liberally choose any arbitrarily large model order for the VAR model with the aim of capturing the full temporal dynamics of fMRI time courses. To objectively select the best order, one could use some information-theoretic criterion. Another practical approach is to first fit a VAR(1); if the autocorrelation plots of the residuals look reasonably like white noise then stop at order 1; if not then continue by fitting a VAR(2) and so on.

The purpose of this paper is to address the aforementioned problems in investigating effective connectivity. Specifically, we aim to extend the VAR model for estimating directed graphs that account for inter-subject variability as well as the high dimensionality of the parameter space. Currently, there is no standard way for this purpose. Here, we develop a novel methodology that puts the VAR model in a Bayesian hierarchical modeling framework that naturally permits modeling sources of variability within and between subjects. We appropriately specify the prior distributions over the parameters of the VAR model in order to achieve an equivalent elastic net penalization approach as developed by Zou and Hastie (2005), and controlling in this way for the large number of parameters and the collinearity in the VAR model regressors (Kyung et al., 2010; Li and Lin, 2010). The modeling strategy also provides a practical Gibbs sampling scheme that is relatively easy to implement.

The remainder of the manuscript is organized as follows. We first formulate our model and develop the Bayesian modeling and inference procedure. This is followed by an application of our proposed method to an fMRI data set collected from a group of participants in a clinical study to determine alterations in effective connectivity due to stroke. We conclude with a discussion that highlights the strengths and advantages of the proposed hierarchical VAR model.

## 2. The hierarchical model for effective connectivity

To investigate effective connectivity, we develop a hierarchical VAR model formulated under the a Bayesian modeling framework. The model has parameters that characterize *experimental condition, group* and *subject-specific* cross-dependence between the *R* ROIs. The key advantages of the proposed hierarchical VAR model are the following: (i) it permits the use of many parameters, which is necessary for characterizing the dependence structure in data derived from complex processes such as fMRI time series, allowing efficient estimation of parameters even in the high dimensional setting and under high multi-collinearity in the regressors, (ii) it quantifies between-subject variations in connectivity, (iii) it identifies Granger causality both at the group and subject level, as well as characterize Granger causality by experimental condition, and (iv) it permits testing for differences in connectivity between patient groups and between experimental conditions.

### 2.1. Single subject multiple-stimuli VAR model notation

In the context of fMRI time series, it is common to register the data according to the timing of the presentation of the stimulus. We allow the VAR coefficients to vary according to the experimental conditions. Moreover, we will assume that following the presentation of condition *A* at time point *t*, the brain effective connectivity for condition *A* is activated and sustained until the future time when condition *B* is presented. Thus, our VAR model has coefficients that change over time (according to the timing of stimulus presentations), but are constant within a local interval until a different condition is presented. We formalize the above ideas by defining the parameters of the VAR model for each condition with the indicator functions *W*^(*s*)^_*A*_(*t*) and *W*^(*s*)^_*B*_(*t*). Suppose that condition *A* was presented at time *t*_1_ and condition *B* at time *t*_1_ + *M*. Thus, from time *t*_1_ to *t*_1_ + *M* − 1, *W*^(*s*)^_*A*_(*t*) takes on the value of 1 and *W*^(*s*)^_*B*_(*t*) takes on the value of 0. At time *t*_1_ + *M*, we have *W*^(*s*)^_*A*_(*t*_1_ + *M*) = 0 and *W*^(*s*)^_*B*_(*t*_1_ + *M*) = 1. Then the VAR model of order *K* for participant *s* with 2 conditions *A* and *B* is defined as

(1) $$Y^{\left(s\right)}(t)=\sum_{k\;=\;1}^{K}\left(\Phi_{A,k}^{\left(s\right)}W_{A}^{\left(s\right)}(t)+\Phi_{B,k}^{\left(s\right)}W_{B}^{\left(s\right)}(t)\right)Y^{\left(s\right)}(t-k)+e^{\left(s\right)}(t)$$

where **Y**^(*s*)^(*t*) = [*Y*^*s*^_1_(*t*), …, *Y*^*s*^_*R*_(*t*)]′, *R* is the number of ROIs, *s* = 1, …, *S*; and *t* = 1, …, *T*_*s*_. The random vector **e**^(*s*)^(*t*) is white noise with 𝔼 **e**^(*s*)^(*t*) = 0 and ℂov **e**^(*s*)^(*t*) = **Σ**_τ_ = diag{τ^−1^_1_, …, τ^−1^_*R*_} does not change over time and it is assumed to be constant across all participants. The subject-specific connectivity parameters are defined by the components of the matrices Φ^(*s*)^_*A, k*_, Φ^(*s*)^_*B, k*_. Under condition *A*, the fMRI time series **Y**^(*s*)^(*t*) follows a VAR model of order *K* and the dependence structures under this condition are summarized in the lag-specific connectivity matrices **Φ**^(*s*)^_*A, k*_ for *k* = 1, …, *K*. For condition *B*, these connectivity matrices are **Φ**^(*s*)^_*B, k*_. The number of mean parameters under the above model is *q* = *R*^2^ × *K* × 2.

In the classical linear model notation, we rewrite the VAR model for participant *s* as,

(2) $$y^{\left(s\right)}=X^{\left(s\right)}\phi^{\left(s\right)}+\varepsilon^{\left(s\right)},\;s=1,\ldots ,S,\;\varepsilon^{\left(s\right)}\sim N(0,\Sigma_{\tau }⊗I_{T-K})$$

where the matrix **X**^(*s*)^ of dimension (*T* − *K*)*R* × *q* is obtained by stacking the *R* × *q* time dependent matrices **x**^(*s*)^(*t*), defined for *t* = *K* + 1, …, *T* as

$$\begin{array}{l} x_{t}^{\left(s\right)}:=I_{R}⊗[W_{A}^{\left(s\right)}(t)Y^{\left(s\right)}{\left(t-1\right)}^{\prime }\ldots W_{A}^{\left(s\right)}(t)Y^{\left(s\right)}{\left(t-K\right)}^{\prime }\\ \;W_{B}^{\left(s\right)}(t)Y^{\left(s\right)}{\left(t-1\right)}^{\prime }\ldots W_{B}^{\left(s\right)}(t)Y^{\left(s\right)}{\left(t-K\right)}^{\prime }], \end{array}$$

so that

$$X^{\left(s\right)}:=\left[\begin{array}{c} x^{\left(s\right)}(K+1)\\ \vdots \\ x^{\left(s\right)}(T) \end{array}\right]\text{ and }y^{\left(s\right)}:=\left[\begin{array}{c} Y^{\left(s\right)}(K+1)\\ \vdots \\ Y^{\left(s\right)}(T) \end{array}\right].$$

### 2.2. Hierarchical structure

To model inter-subject variability and derive inferences at the subject and group level, we describe the subject-specific vector of parameters as **ϕ**^(*s*)^ = **ϕ** + **ξ**^(*s*)^, where **ϕ**^(*s*)^ is a vector of dimension *q* = *R*^2^ × *K* × 2, defined as,

(3) $$\phi^{\left(s\right)}:=\text{vec}\left[\begin{array}{c} \Phi_{A,1}^{\left(s\right)}\ldots \Phi_{A,K}^{\left(s\right)}\Phi_{B,1}^{\left(s\right)}\ldots \Phi_{B,K}^{\left(s\right)} \end{array}\right].$$

The vector **ξ**^(*s*)^ represents the subject-specific deviations such that **ξ**^(*s*)^ | **D** ~ *N*_*q*_(0, **D**), and **D** = diag{*d*^ − 1^_1_, *d*^−1^_2_, …, *d*^−1^_*q*_}. The elements of **D** quantify the variability between subjects in the connectivity parameters.

Because of the subject-specific parameters in the model, the covariance matrix also varies across subjects, and in fact, it is completely determined by the subject-specific coefficients **ϕ**^(*s*)^ and the noise covariance **Σ**_τ_ = diag{τ^−1^_1_, …, τ^−1^_*R*_}. To show this, adopting the matrix notation of connectivity parameters established in Equation 1, first we define **M**_*s*_: = [Φ^(*s*)^_*A, k*_*W*^(*s*)^_1_(*t*) + Φ^(*s*)^_*B, k*_*W*^(*s*)^_2_(*t*)]. Then we rewrite the model as,

$$Y^{\left(s\right)}(t)=M_{s}Y^{\left(s\right)}(t-1)+e^{\left(s\right)}(t)$$

where, 𝔼(**e**^(*s*)^(*t*)) = 0, ℂov (**e**^(*s*)^(*t*), **e**′^(*s*)^(*t*)) = **Σ**_τ_, and ℂov(**e**^(*s*)^(*t*), **e**′^(*s*)^(*k*)) = 0. Therefore, following the procedure detailed in Lütkepohl (2005), the subject-specific covariance matrix is given by

(4) $$\mathbb{C}\text{ov}(Y^{\left(s\right)}(t))=\sum_{i=0}^{\infty }M_{s}^{i}\Sigma_{\tau }M_{s}^{i\prime }$$

where the matrix **M**^*i*^_*s*_ represent **M** to the *i*-th power and it is absolutely summable under the vector autoregressive framework. From Equation (4), now we can see clearly that the covariance matrix varies across subjects, which is a consequence of the subject-specific coefficients.

The priors over the group connectivity parameters **ϕ** are defined as suggested in Kyung et al. (2010) and Li and Lin (2010) to achieve the equivalent elastic net estimators. The proposed prior distribution is

(5) $$\phi |V_{\tau },V_{\Phi }\sim N(0,V_{\tau }V_{\Phi }),$$

for **V**_τ_ := diag{[τ^−1^_1_ **1**_2*RK*_ … τ_*R*_^−1^**1**_2RK_]} where **1**_2*RK*_ is a vector of ones with dimension 1 × 2*RK* and τ_*r*_ ~ Gam(*r*_τ_*r*__, *h*_τ_*r*__); **V**_Φ_ := diag{(α_1_ + λ_2_)^−1^, (α_2_ + λ_2_)^−1^, …, (α_*q*_ + λ_2_)^−1^} and α_*j*_ | λ_2_, γ ~ (α_*j*_/(α_*j*_ + λ_2_))^1/2^ InvGamma(1, γ/2), where λ_2_ ~ Gam(*r*_λ_, *h*_λ_) and γ ~ Gam(*r*_γ_, *h*_γ_).

There is a subset of parameters in the vector **ϕ** linked to the variance τ^−1^_*r*_ via the definitions of **V**_Φ_ and **V**_τ_. Let **ϕ**_ (*r*)_ := {ϕ_*j*_ : ϕ_*j*_ ~ *N*(0, τ^−1^_*r*_(α_*j*_ + λ_2_)^−1^)} be the subset of parameters associated with τ_*r*_. For **ϕ**_(*r*)_, the corresponding normal distribution given in Equation (5) is equivalent to $\phi_{\left(r\right)}|\tau_{r}\propto \exp\left\{-\frac{1}{2}\left(2\sqrt{\gamma \tau_{r}}|\phi_{\left(r\right)}|+\lambda_{2}\tau_{r}||\phi_{\left(r\right)}|{|}^{2}\right)\right\}$, and this prior distribution is equivalent to the elastic net approach commonly used for variable selection (Li and Lin, 2010). Thus, for the vector **ϕ**, we have the prior distribution $\phi |V_{\tau }\propto \exp\left\{-\frac{1}{2}\left(2\sqrt{\gamma }|\sqrt{V_{\tau }^{-1}}\phi |+\lambda_{2}||V_{\tau }^{-1}\phi |{|}^{2}\right)\right\}$, which is equivalent to the elastic net.

**Figure 1:**
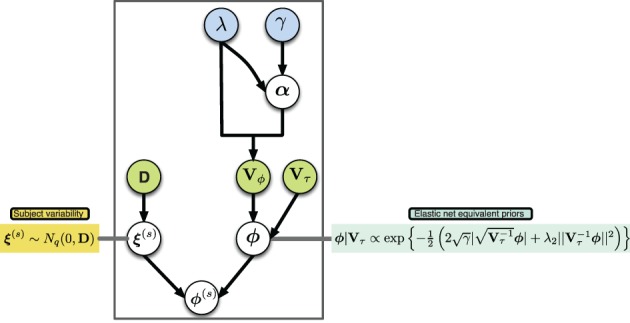
**Graphical model of proposed hierarchical structure, arrows represent parameter dependence, blue fill indicates equivalent penalization parameters in the elastic net setting, green fill indicates matrices with parameters of variance**.

A graphical description of the hierarchical structure is presented in (Figure 1). Note that even though we shrink the group connectivity parameters toward zero via their conditional priors, we allow the subject specific connectivity parameters to be different from zero per the subject-specific deviations **ξ**^(*s*)^. In this way we impose a sparse structure at the group level while simultaneously allowing for subject-specific deviations from this structure.

Now we describe the full conditional distributions that we used in our Gibbs sampler. First, the structure of the posterior distribution of the model is

(6) $$\begin{array}{l} \prod_{s=1}^{S}N_{y}(X^{\left(s\right)}\phi +Z^{\left(s\right)}\xi^{\left(s\right)},\Sigma_{\tau }⊗I_{T-K})N_{\phi }(0,V_{\tau }V_{\Phi })\\ \;N_{\xi }(0,D)\prod_{j=1}^{q}\text{Gam}(r_{d_{j}},h_{d_{j}}) \end{array}$$

$$\begin{array}{l} \overset{R}{\underset{n=1}{\prod }}\text{Gam}(r_{\tau_{n}},h_{\tau_{n}})\overset{q}{\underset{j=1}{\prod }}{\left(\alpha_{j}/\left(\alpha_{j}+\lambda_{2}\right)\right)}^{1/2}\\ \;\;\;\;\;\;\text{InvGa}{\text{m}}_{\alpha_{j}}(1,\gamma /2)\text{Gam}(r_{\lambda },h_{\lambda })\text{Gam}(r_{\gamma },h_{\gamma }). \end{array}$$

To describe the conditional distributions for the Gibbs sampling, we will use the following matrix notation obtained by stacking the vectors and matrices stated in Equation (2):

$$\begin{array}{l} y=\left(\begin{array}{c} y^{\left(1\right)}\\ y^{\left(2\right)}\\ \vdots \\ y^{\left(S\right)} \end{array}\right),\;X=\left(\begin{array}{c} X^{\left(1\right)}\\ X^{\left(2\right)}\\ \vdots \\ X^{\left(S\right)} \end{array}\right),\;Z=\left(\begin{array}{llll} Z^{\left(1\right)} & 0 & \ldots & 0\\ 0 & Z^{\left(2\right)} & \ldots & 0\\ \vdots & \vdots & \ddots & \vdots \\ 0 & 0 & \ldots & Z^{\left(S\right)} \end{array}\right),\\ \xi =\left(\begin{array}{c} \xi^{\left(1\right)}\\ \xi^{\left(2\right)}\\ \vdots \\ \xi^{\left(S\right)} \end{array}\right),\;D^{*}=\left(\begin{array}{llll} D & 0 & \ldots & 0\\ 0 & D & \ldots & 0\\ \vdots & \vdots & \ddots & \vdots \\ 0 & 0 & \ldots & D \end{array}\right),\\ I_{\tau }=\Sigma_{\tau }⊗I_{S(T-K)}. \end{array}$$

From Equation (6), we obtain next the full conditional distributions:

- *group effective connectivity* **ϕ** ~ *N* (**μ**_**ϕ**_,**V**), where
  $$\begin{array}{l} \mu_{\phi }={\left(X^{\prime }I_{\tau }^{-1}X+V_{\tau }^{-1}V_{\phi }^{-1}\right)}^{-1}X^{\prime }I_{\tau }^{-1}(y-Z\xi ),\text{ and}\\ V={\left(X^{\prime }I_{\tau }^{-1}X+V_{\tau }^{-1}V_{\phi }^{-1}\right)}^{-1}; \end{array}$$
- *subject-specific deviations* **ξ** ~ *N*(**μ**_**ξ**_, **V**_**ξ**_), where
  $$\begin{array}{l} \mu_{\xi }={\left(Z^{\prime }I_{\tau }^{-1}Z+D^{*-1}\right)}^{-1}Z^{\prime }I_{\tau }^{-1}(y-X\phi ),\text{ and}\\ V_{\xi }={\left(Z^{\prime }I_{\tau }^{-1}Z+D^{*-1}\right)}^{-1}; \end{array}$$
- *the variance components of* **ϕ**
  $$\begin{array}{l} \alpha_{j}\sim \text{InvGauss}\left(\gamma ,\sqrt{\frac{\gamma }{\tau_{h(j)}\phi_{j}^{2}}}\right),\;\tau_{h(j)}:=V_{\tau }^{-1}(j,j)\\ \tau_{r}\sim \text{Gamma}(\frac{(T-K)S+2RK+2r_{\tau_{r}}}{2},\frac{1}{2}w_{{\left(r\right)}^{\prime }}w_{\left(r\right)}+\frac{1}{2}\zeta_{{\left(r\right)}^{\prime }}\zeta_{\left(r\right)}+h_{\tau_{r}}) \end{array}$$
  where
  $$\begin{array}{l} w_{\left(r\right)}:=\{w_{i}\in w:I_{\tau }(i,i)=\tau_{r}^{-1}\},\\ \;\;\;w:={\left(y^{\prime }-X\phi -Z\xi^{\prime }\right)}^{\prime }\left(y^{\prime }-X\phi -Z\xi^{\prime }\right) \end{array}$$
  with
  $$\begin{array}{l} \zeta_{\left(r\right)}:=\{\zeta_{j}\in \zeta :V_{\tau }(j,j)=\tau_{r}^{-1}\}\;\zeta :=\phi^{\prime }V_{\phi }^{-1}\phi ,\\ \lambda_{2}\sim \text{Gamma}\left(r_{\lambda }+\frac{q}{2},\frac{1}{2}\sum_{j=1}^{q}\tau_{h(j)}\phi_{j}^{2}+h_{\lambda }\right),\text{ and}\\ \gamma \sim \text{Gamma}\left(r_{\gamma }+q,\frac{1}{2}\sum_{j=1}^{q}\alpha_{j}^{-1}+h_{\gamma }\right); \end{array}$$
- *the variance components of* **ξ**:
  $$d_{j}\sim \text{Gamma}\left(S(r_{d}-1/2)+1,\;\frac{1}{2}\sum_{s=1}^{S}{\left(\xi_{j}^{\left(s\right)}\right)}^{2}+Sh_{d}\right),$$
  this for the case of having set the prior distribution of *d*_*j*_ as *d*_*j*_ ~ Gamma(*r*_*d*_, *h*_*d*_).

The parameters in all Gamma distributions correspond to the parameters of shape and rate respectively.

### 2.3. Effective connectivity networks

#### 2.3.1. Group-specific effective connectivity networks

We infer a group-specific Granger causality network by selecting the parameters whose estimates survive some significance threshold, in our case we use the 95% credible region criteria. To construct the group-specific Granger causality network for experimental condition *A*, we draw a directed edge from region *r* to a region *r*′ to representing Granger-causality if the 95% credible region of the lag-joint parameter distribution of [**Φ**^(*s*)^_*A*, 1_(*r*′, *r*), …, **Φ**^(*s*)^_*A, K*_(*r*′, *r*)] does not include the origin. In the case of a VAR model of order 2, a credible region from the posterior samples at lag 1 and lag 2 of a specific connectivity parameter can be obtained by constructing an empirical 3-dimensional histogram, and a 95% credible region is defined by the 0.025 contour level line of this histogram.

To compare Granger causality across two experimental conditions *A* and *B*, at each link from region *r* to *r*′, we calculate the differences per lag, **Φ**^(*s*)^_*A*, 1_(*r*′, *r*) − **Φ**^(*s*)^_*B*, 1_(*r*′, *r*), …, **Φ**^(*s*)^_*A, K*_(*r*′, *r*) − **Φ**^(*s*)^_*B, K*_(*r*′, *r*) and declare a difference in Granger causality to be significant when the credible regions associated to the lag-joint parameter distribution of the differences do not include the origin. Similarly, we compare Granger causality across groups from the lag-joint parameter distribution of the differences between groups.

#### 2.3.2. Subject-specific effective connectivity networks

To construct a subject-specific Granger causality network, we use the individual connectivity coefficients determined by the group connectivity coefficients plus the subject deviation: **ϕ**^(*s*)^ = **ϕ** + **ξ**^(*s*)^. We then proceed to build the network in a manner that is analogous to that at the group level. Our approach to test for differences in Granger causality at the subject level between experimental conditions is similar to the approach at the group level.

#### 2.3.3. Subject variability over connectivity coefficients

We quantify the variance among connectivity coefficients via the parameters diag{*d*^−1^_1_, *d*^−1^_2_, …, *d*^−1^_*q*_}. Specifically, the parameter *d*^−1^_*j*_ represents the variance among subjects for the corresponding connectivity coefficient. Where the correspondence is imposed by the order given in Equation (3). If the variance is very small relatively to the population value of **Φ**_*Cond.,k*_(*r*′, *r*), then we can say that the variations across subjects for the associated link can be ignored.

#### 2.3.4. Modeling multi-group effective connectivity

If we have several groups in the analysis, we can state the proposed model to allow for group-specific connectivity parameters. To illustrate, suppose we have two groups and each group has its own connectivity parameters. Let **ϕ** := [**ϕ**_Grp.1_ **ϕ**_Grp.2_] where **ϕ**_Grp.*j*_ are the connectivity coefficients for group *j*, **ϕ**_Grp.*j*_ := vec[**Φ**_*A*, 1_ … **Φ**_*A, K*_ **Φ**_*B*,1_ … **Φ**_*B, K*_]. To include each group's own connectivity coefficients, we re-define the matrices **X**, D^*^ as

$$\begin{array}{l} X=\left(\begin{array}{c} X^{\left(1\right)}A^{\left(1\right)}\\ X^{\left(2\right)}A^{\left(2\right)}\\ \vdots \\ X^{\left(S\right)}A^{\left(S\right)} \end{array}\right),\;D^{*}=\left(\begin{array}{cccc} A^{\left(1\right)}D & 0 & \ldots & 0\\ 0 & A^{\left(2\right)}D & \ldots & 0\\ \vdots & \vdots & \ddots & \vdots \\ 0 & 0 & \ldots & A^{\left(S\right)}D \end{array}\right),\text{ and}\\ D=\left(\begin{array}{c} \begin{array}{l} D_{\text{Grp}.1}\\ D_{\text{Grp}.2} \end{array} \end{array}\right) \end{array}$$

where **A**^(*s*)^ = [**I**_*q*_ **0**] if subject *s* belongs to group 1, **A**^(*s*)^ = [**0 I**_*q*_] if subject *s* belongs to group 2, and **D**_Grp.*j*_ is the diagonal matrix that quantifies the inter-subject variability among subjects from group *j*. Therefore, the conditional posterior distributions of the parameters in **D**_Grp.*j*_ are slightly modified by considering the subjects in group *j*.

## 3. Effective connectivity analysis in a fMRI stroke study

To demonstrate the utility of the proposed model, we analyzed functional magnetic resonance imaging (fMRI) time series data collected from healthy controls and stroke patients with residual motor deficit. Chronic stroke is associated with well-documented bilateral changes in fMRI activation strength and volume within the motor system. However, it is possible that there may be stroke-related differences in connectivity both with and without a stimulus that are not equally reflected in changes in fMRI activation strength or volume (Rehme and Grefkes, 2013). Our goal in this paper is to develop a flexible statistical model for investigating these contrasts in effective connectivity.

### 3.1. Participants

Entry criteria include age ≥ 18 years, ischemic stroke 11–26 weeks prior to first study assessments, and some residual motor deficit—Action Research Arm Test score <52 (range = 0–57, normal = 57) OR 9 hole-peg test score <25% of the unaffected hand score. In this analysis, there were two groups: 16 stroke patients with the right side of their body affected and 13 healthy participants that served as control group.

### 3.2. Tasks, apparatus and procedures

A Philips Achieva 3.0T MRI whole-body scanner was used to collect patients' imaging data. High-resolution T1-weighted images were acquired using a 3D MPRAGE sequence (repetition time (*TR*) = 8.5 ms, echo time (*TE*) = 3.9 ms, flip angle = 8, field of view (FOV) = 256× 204 × 150, slices = 150, voxel size = 1 × 1 × 1 mm^3^). Blood oxygenation level-dependent (BOLD) images were acquired using a T2^*^-weighted gradient-echo echo planar imaging sequence (*TR* = 2000 ms, *TE* = 30 ms, flip angle = 70, FOV = 240 × 240 × 154, slices = 31, voxel size = 2 × 2 × 2 mm^3^). The MRI protocols were the same for each patient. Functional magnetic resonance imaging (fMRI) contrasted affected hand grasp-release movements (active condition) with rest condition.

For the participants in the healthy control group, the experiment was divided into 2 sessions each with 48 fMRI scans, alternating 12 scans between the two conditions, always starting with rest condition. Therefore the total time series points that were considered in the analysis for each participant in this group is *T* = 2× 48 = 96. For participants within the right side affected group, the experiment was divided into 3 sessions each with 48 fMRI scans, alternating 12 scans between the two conditions but always starting with rest condition. Therefore the total time series points that were considered in the analysis for each participant in this group is *T* = 3× 48 = 144.

### 3.3. Selection of regions of interest

**Figure 2:**
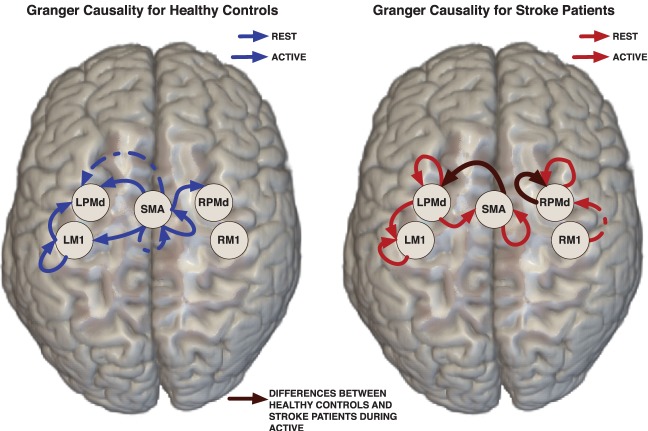
**Links represent Granger causality connections determined by the 95% credible regions defined by the 0.025 contour level of the lag-joint sample distribution of connectivity coefficients on each group**. Brown edge is significantly different between healthy control group and stroke group in the Granger causality networks during active condition.

Using the Marsbar toolbox (Brett et al., 2002), bilateral ROIs were created within primary motor cortex (M1) and dorsal premotor cortex (PMd), and a midline supplementary motor area (SMA) ROI. The mean coordinates of the ROIs were taken from a meta-analysis by Mayka et al. (2006). Each of these regions was defined by a 6.0 mm radius sphere around the specific local coordinates. We summarized the information across the 110 voxels in each ROI by the mean of fMRI time series. We consider the mean time course within the ROI is a good representative of the BOLD activity in each specified ROI because the images were smoothed during the preprocessing step (as described below). The ROIs constructed for the analysis are small—spheres with a radius of 6 mm—relative to the Gaussian smoothing kernel having FWHM of 8 mm used to smooth the images. Thus, the neighboring voxels within each ROI in the smoothed image are similar to each other. The ROIs considered in our analysis are localized as in (Figure 2). To illustrate the proposed model, we focus only on these 5 preselected regions which are known to be highly involved in the task experiment after stroke.

### 3.4. Preprocessing steps

Functional data from all the sessions were preprocessed using SPM8 software (Wellcome Trust Center for Neuroimaging, www.fil.ion.ucl.ac.uk/spm). Preprocessing steps included realignment to the first image, coregistration to the mean EPI image, normalization to the standard MNI EPI template, and spatial smoothing (FWHM = 8 mm). Data was visually inspected for head movement. Data was rejected for patients with >2 mm head displacement.

Previous to the VAR model fitting, we removed expected trends on averaged fMRI time series from each ROI and each participant. The expected trends were obtained per subject and per ROI by fitting the linear model with the following regressors: (1) the expected BOLD condition-specific response *X*_*c*_(*t*), defined as the convolution between the canonical hemodynamic response function (difference of two gamma functions) and the indicator function for condition *c* (in our case, rest and active); (2) drift components which were defined as polynomials up to the order three; and (3) seasonal components (sines and cosines at frequencies below 0.25 Hz and above 0.5 Hz) to remove cardiac and respiratory effects. To analyze connectivity, the proposed hierarchical VAR model was fit to the ROI-specific and subject-specific residuals which were obtained by removing the estimated from the observed average fMRI time series.

### 3.5. Time series for the proposed hierarchical connectivity model

Our data consists of subject-specific multivariate time series (with 5 components representing brain activity in the selected 5 ROIs) from two groups: 13 participants in the healthy control group and 16 patients in the stroke group. The length of time series in the control group is 96, while in the stroke group is 144. Note that fitting a VAR model of order (maximal lag) *K* having connectivity parameters representing each condition (rest/active) requires a total of 2× *K* × 25 parameters per group (control/stroke), plus the parameters that describe the individual deviations from the group. Therefore in this case, there is a need to control for the large number of parameters to be considered in the model. We fit the proposed hierarchical VAR model of order *K* = 2 while accounting for the large number of parameters via elastic net. The first order VAR model, is often recommendable for fMRI time series, given the low temporal resolution on this data. There are a number of papers that suggest a VAR order 1 can sufficiently capture the covariance structure in a multi-ROI fMRI data (Martínez-Montes et al., 2004; Valdes-Sosa et al., 2005). Here, we first fit the VAR(1) model but the diagnostics (based on the auto-correlation of the residuals) suggest that it does not sufficiently capture the temporal dynamics. As a next step, we fit a VAR(2) model which turned out to be adequate for the stroke data at hand. The model was implemented by a Gibbs sampling procedure; there were generated 80000 iterations. The first 60000 iterations were discarded. For inference purposes, the last 20,000 posterior samples were thinned by sampling every 5-th sample to get the posterior distribution sample. We investigated the convergence of the samples by inspecting trace plots and via the Geweke technique as described in Brooks and Roberts (1998), we do not find evidence of lack of convergence.

### 3.6. Results of effective connectivity analysis

#### 3.6.1. Group-specific granger causality networks

Effective connectivity group-networks were constructed in terms of the Granger causality concept. To identify Granger causality links, we consider the 95% level credible regions defined by the lag-joint distribution of VAR-coefficients of each group and we select the links associated to the regions that did not include the origin. Credible regions were delimited by the 0.025 contour line of the empirical histogram that approximates the lag-joint distribution. (Figure 2) shows the Granger causality networks by group and condition. In both groups, more connectivity links were observed in the active phase than during rest. During movement there is up-regulation of areas within the motor system, compared to rest, due to the internally driven processes required to move as well as the afferent feedback resulting from execution of the movement. The degree of unilateral connectivity is an observable difference between healthy control and stroke groups. As would be expected in non-injured brains, the control group exhibited connectivity patterns during movement that were largely confined to the hemisphere contralateral to movement. However, in the stroke group, additional connections were also observed in the contralesional hemisphere, specifically contralesional PMd. This is in-line with previous studies that report widespread activation in the contralesional hemisphere, including contralesional PMd, during movement of the paretic hand (Rehme et al., 2012). Indeed, contralesional PMd has been to shown to play a compensatory role in paretic arm movement (Johansen-Berg et al., 2002; Bestmann et al., 2010).

#### 3.6.2. Network differences between control and stroke groups

We determined the significant differences in Granger causality between the healthy control group and stroke patients; these differences are indicated with brown links in (Figure 2). In particular we found a difference during the active condition in the influence of activity in the SMA on activity in the left PMd. In healthy controls, SMA activity predicted the subsequent activity in left PMd during movement of the right hand. In contrast, this connectivity link was not observed in the stroke patients. This result supports prior studies that indicate a reduction in ipsilesional intrahemispheric connectivity after stroke (Rehme and Grefkes, 2013).

For the stroke group, our findings of no significant connectivity between SMA and ipsilesional PMd may be related to a reduction of top-down control of motor movement in the impaired limb by the damaged hemisphere in patients with persisting post-stroke motor impairments. Dorsal premotor cortex is known to be important for higher order functions involved in motor planning (Rushworth et al., 2003; O'Shea et al., 2007; Wu and Hatsopoulos, 2007; Nakayama et al., 2008; Chouinard and Paus, 2010). Meanwhile, SMA has been implicated in internally generated tasks (Tanji, 1994; Chen et al., 2009), bimanual coordination (Serrien et al., 2002; Steyvers et al., 2003), and programming of movements prior to execution (Roland et al., 1980). As in the present study, Sharma et al. (2009) also found a significant reduction in connectivity between SMA and ipsilesional premotor cortex in patients compared to controls during both paretic hand movement and motor imagery (Sharma et al., 2009). However, a prior study found that there were no significant connectivity differences during movement between healthy controls and patients 3–6 months post stroke (Rehme et al., 2011). Although not statistically significant, they did see a connection between contralateral SMA and PMd that was not present in patients at 3–6 months post-stroke. This non-significant difference was also observed in a study of patients with subcortical stroke (Grefkes et al., 2008). The mixed findings may be attributable to the different effective connectivity models used in the analyses. Additional studies are needed to resolve the inconsistent findings within the limited post-stroke fMRI effective connectivity literature.

Comparing the region-specific model variances between the stroke patients and the healthy controls, we identified an increase in the variability for the ROIs in the patient control group relative to the healthy group, the region specific variances are given in (Table 1)

**Table 1:** **Summary of posterior region-specific variances for the error term in the hierarchical model**.

**Group**	**ROI**	**Q.025**	**Q.50**	**Q.975**
Healthy controls	LM1	5.5124	5.988	6.5183
	LPMd	5.3635	5.8234	6.3431
	RM1	4.5715	4.9502	5.3803
	RPMd	6.1512	6.7013	7.2669
	SMA	6.2479	6.7828	7.4008
Stroke patients	LM1	12.985	13.795	14.676
	LPMd	13.369	14.173	15.064
	RM1	11.238	11.929	12.69
	RPMd	9.2354	9.8209	10.445
	SMA	9.7889	10.383	11.055

*Defined as the unique diagonal elements in the matrix V_τ_*.

#### 3.6.3. Between-subject variation in effective connectivity

**Figure 3:**
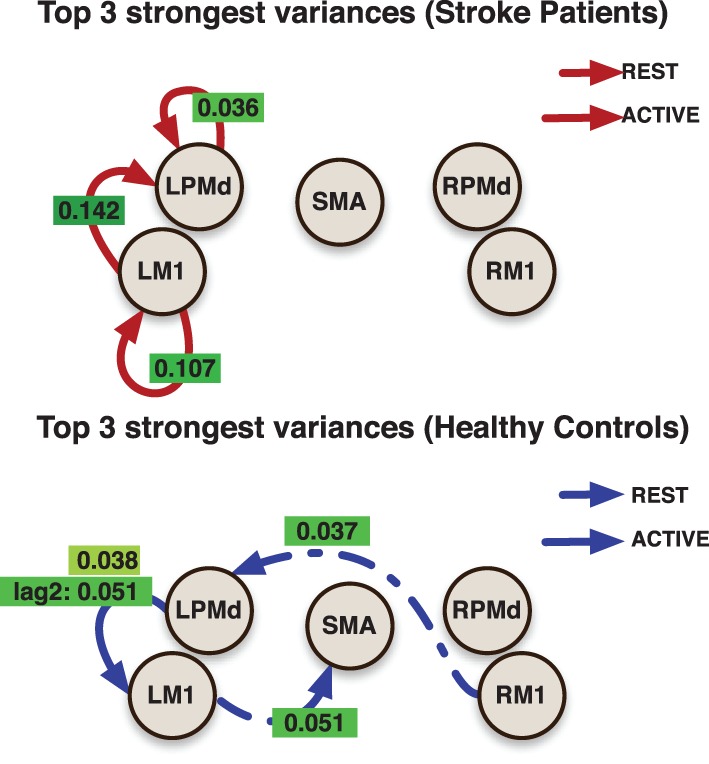
**Links indicate the top 3 largest inter-subjects variances over connectivity coefficients for each group**. Labels over links show the posterior mean value for the variance associated to the link. All variances represent the effect from the previous value (with the exception of the labeled as lag 2) in the indicated region to current value at the target region.

**Figure 4:**
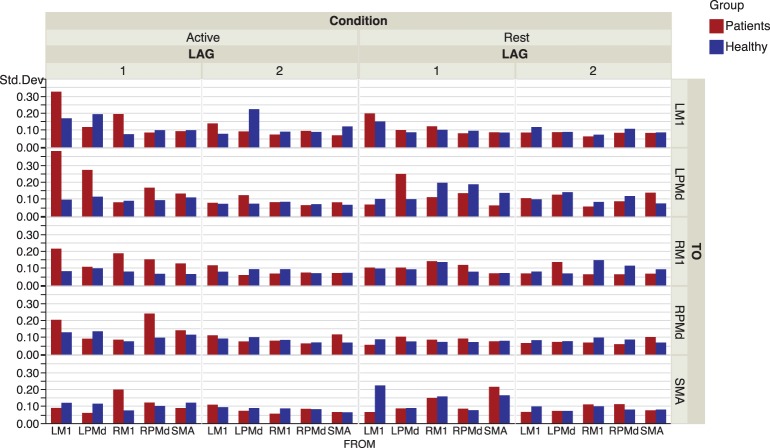
**Inter-subject standard deviations over connectivity coefficients for each group and condition**.

The effective connectivity model also identified specific connections that accounted for the greatest variability within groups. (Figure 3) presents the edges over which the top three largest group variances associated to the coefficients occurred. Large variability was also present in the motor network during the active phase. An overall picture of between subject standard deviations for all the considered links is presented in (Figure 4).

**Figure 5:**
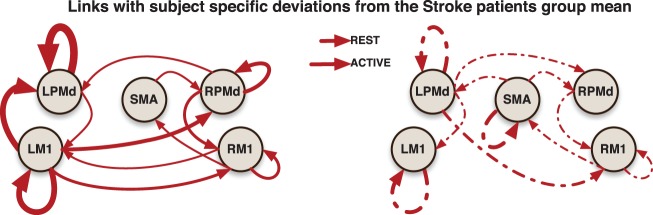
**Links indicate that at least one significant subject-specific deviation was found on that connection**. The thicker the link, the more number of significant deviations were identified on it.

In addition, more significant deviations were found in the patient group. This behavior could be explained by both the presence of infarcts (an irregularity that is in the stroke but not patient groups) and the randomness in the sites of these infarcts. A general picture of the links at which these subject specific deviations were identified is presented in (Figure 5).

**Figure 6:**
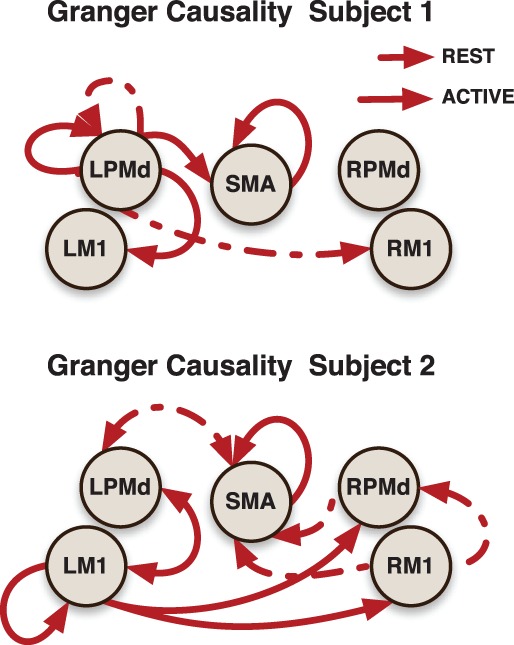
**Granger causality subject-networks for two subjects in the stroke patients group**. Links were determined by 95% credible regions of subject specific posterior means at lag 1 and 2.

Subject-specific Granger causality networks can be constructed in a similar way to the group network by analyzing the individual prediction parameters established in the model. To illustrate this point, we present two Granger causality networks for two patients in the stroke group (Figure 6). Connectivity maps from these two patients did show reciprocal links between ipsilesional M1 and contralesional motor areas that were not seen at the group level. This is a connection that pervades the post-stroke connectivity literature. Stroke has been associated with increased inhibition from contralesional M1 onto ipsilesional M1, as well as reduced inhibition of contralesional M1 by ipsilesional M1 (Murase et al., 2004; Rehme and Grefkes, 2013). A prior effective connectivity study did find that, compared to controls, stroke patients exhibited a negative influence of contralesional M1 to ipsilesional M1 during right hand movement (Grefkes et al., 2008). Considering the heterogeneity of the patient group, a larger study and a model that takes into account subject-specific anatomical information would improve statistical power and could resolve this finding.

## 4. Conclusion

We proposed the hierarchical VAR model to: (i) determine group and condition specific effective connectivity networks; (ii) compare connectivity between patient groups and between conditions; and (iii) investigate subject specific deviations from the network group. To control for the large parameter space and high correlation between the covariates of the VAR model, we used prior distributions so that the estimates are equivalent to that obtained from the elastic net penalization approach.

Because our prior distributions are equivalent to the elastic net penalization, our proposed model allows one to explore richer dependence structures (for example, fitting a VAR model of higher order) than the typical approach of using frequentist methods for fitting VAR models. This is a tremendous advantage especially when we do not have a large sample size such as the case of short fMRI time series. Even though our proposed modeling approach will regularize the parameter estimates in case the dimensionality of the parameter space is large, we still advise caution with the use of an unreasonably large number of ROIs and model order, recalling that the number of parameters for each subject grows quadratically with the number of ROIs and linearly with the model order. As a practical implementation, we suggest fitting a VAR(1) first and then proceed with model diagnostics on the residuals. If the residuals appear like white noise (via auto-correlation plots) then a higher order is not necessary; otherwise, proceed to fitting a VAR with higher order and check the residuals.

Another important issue is the choice of a “representative” time series within a ROI. Here, we used the mean time course within the ROI which is the most common approach. However, there are other potential approaches that we bring up for discussion. First, one could build another level in the hierarchical structure so that the resulting hierarchy becomes: time series per voxel; *voxels within* ROIs; ROIs within a subject; subjects within a patient group. One could them introduce a local within-ROI spatial covariance structure using the ideas in Bowman (2005) and Kang et al. (2012). This procedure could of course introduce another layer of computational complexity. We did not pursue this approach here because the volumes of each ROI in our analysis was small and the fMRI time series at voxels within each ROI are highly correlated, and as a result, any additional information gained will be small in comparison to the computational expense. A second alternative is to perform some dimension reduction step say, via principal components analysis, which will result in a few number of time series within an ROI that accounts for, say 80%, of the total variability within the ROI. A third alternative is to use summaries that are robust to outliers. Some examples include the median or trimmed means. A rigorous study that compares the current approach against these three alternatives is beyond the scope of this paper.

The number of studies using effective connectivity to study post-stroke motor networks are relatively limited compared to traditional fMRI studies. Some key findings thus far include, increased connectivity from the prefrontal cortex within the ipsilesional hemisphere, reduced inhibition of the ipsilesional hemisphere onto contralesional M1, and various excitatory increases/decreases within the ipsilesional hemisphere. The proposed VAR model elucidated the following: (1) specific differences in patterns of effective connectivity between the chronic stroke and healthy control groups; and (2) quantification of between-subject variability in effective connectivity. These results could not have been derived from either DCM, SEM, or standard VAR models. The proposed model could be extended to include covariates (such as the volume of the infarct, amount of overlap between the stroke region and the cortico-spinal tract) and thus could potentially help to identify biomarkers of motor function improvements with post-stroke therapies.

### Conflict of interest statement

The authors declare that the research was conducted in the absence of any commercial or financial relationships that could be construed as a potential conflict of interest.
